# Energy-Efficient Heterogeneous Wireless Sensor Deployment with Multiple Objectives for Structural Health Monitoring

**DOI:** 10.3390/s16111865

**Published:** 2016-11-06

**Authors:** Chengyin Liu, Zhaoshuo Jiang, Fei Wang, Hui Chen

**Affiliations:** 1 Department of Civil and Environmental Engineering, Harbin Institute of Technology Shenzhen Graduate School, Shenzhen 518055, China; chengyin.liu@hitsz.edu.cn (C.L.); hui_chen2015@163.com (H.C.); 2 School of Engineering, San Francisco State University, San Francisco, CA 94132, USA; 3 Department of Electronic and Information Engineering, Harbin Institute of Technology Shenzhen Graduate School, Shenzhen 518055, China; wangfei@hitsz.edu.cn

**Keywords:** heterogeneous wireless sensor networks, modal information quality, energy consumption, clustering

## Abstract

Heterogeneous wireless sensor networks (HWSNs) are widely adopted in structural health monitoring systems due to their potential for implementing sophisticated algorithms by integrating a diverse set of devices and improving a network’s sensing performance. However, deploying such a HWSN is still in a challenge due to the heterogeneous nature of the data and the energy constraints of the network. To respond to these challenges, an optimal deployment framework in terms of both modal information quality and energy consumption is proposed in this study. This framework generates a multi-objective function aimed at maximizing the quality of the modal information identified from heterogeneous data while minimizing the consumption of energy within the network at the same time. Particle swarm optimization algorithm is then implemented to seek solutions to the function effectively. After laying out the proposed sensor-optimization framework, a methodology is present to determine the clustering of the sensors to further conserve energy. Finally, a numerical verification is performed on a four-span pre-stressed reinforced concrete box-girder bridge. Results show that a set of strategically positioned heterogeneous sensors can maintain a balanced trade-off between the modal information accuracy and energy consumption. It is also observed that an appropriate cluster-tree network topology can further achieve energy saving in HWSNs.

## 1. Introduction

Structural health monitoring (SHM) has been widely used to monitor and diagnose the health status of civil structures/infrastructures in real-time [1]. Typically, various sensors are deployed on critical locations of the structure to periodically collect different types of data relevant to the health status. For example, to monitor a bridge, engineers deploy sensors such as accelerometers, strain gauges, and displacement transducers. The collected data will be post-processed according to time/frequency domain algorithms to assess structural conditions, after which the SHM system will estimate the residual life of the structure and, possibly, send out alerts when it exceeds some pre-defined threshold.

Traditional SHM systems rely on wired sensors. The installation and maintenance cost of these sensors represents a large portion of the total cost of the system. With the advances in technology, the development of wireless sensor technology in recent years offers new opportunities for SHM applications [2,3,4,5]. Wireless communication eliminates the cost of wiring and increases the scalability of the SHM systems. Wireless sensor nodes are generally powered by batteries and mounted with multiple sensing devices placed inside and around the structure to periodically collect various types of data. Consequently, wireless sensor networks (WSNs) particularly designed for SHM purpose are evolving into heterogeneous systems [6]. Among various advantages, for example, heterogeneous wireless sensor networks (HWSNs) can potentially provide higher levels of computational power, network densities and lifetimes [7].

As a large amount of existing structures are suffering from deterioration, excessive loadings and unpredicted incidents, the development of dense, yet low-cost wireless sensor arrays to monitor the operation of structural systems has become an attractive research subject in recent years. Some notable experimental applications implemented on large-scale structures can be seen, for instance, in the Golden Gate Bridge [8] and the Jindo Bridge [9,10] as well as in several other long-span [11,12] and medium-length span bridges [13,14]. However, WSNs are not yet commonly adopted in permanent monitoring systems largely due to the challenges of providing necessary power for the monitoring system through the limited power supply of sensor nodes with relatively small size. Thus, WSNs are still considered as less popular alternatives to conventional wired SHM systems. Therefore, maintaining an optimal size of sensor network that can provide the desired modal information is deemed as one of the most critical challenges in the current deployment practice of WSNs in SHM.

In this paper, a study attempting to implement optimal sensor deployment (OSD) techniques to determine the number and locations of heterogeneous wireless sensors on a structure of interest has been performed. This implementation of such system intends to produce reliable information about the structure’s health status while maintaining reasonable energy consumption.

As an important issue in SHM, the OSD techniques have been demonstrated to be effective in helping to achieve accurate estimation of modal parameters by placing the sensors properly. Within the context of OSD, quite a few methods, such as the Effective Influence (EI) method [15], Modified Variance (MV) method [16], and Kinetic Energy (KE) method [17], have been proposed and verified by practical SHM implementations. In addition to these existing OSD techniques designed for modal parameter estimation in wired SHM systems, a handful of works have attempted to address the OSD issues for wireless sensor networks. For instance, Bhuiyan et al. [18,19] proposed a three-phase sensor placement method for SHM that addressed the quality of sensor placements, communication efficiency, and fault tolerance. Onoufriou et al. [20] presented a two-step strategy to optimize the number of sensors and their locations to satisfy both specific structural engineering requirements and energy constraint imposed by a WSN. Zhou et al. [21] formulated an energy-aware wireless sensor placement framework and developed a hybrid discrete firefly algorithm to solve complex optimization problem. Fu et al. [22] performed a study to optimize wireless sensor placement for SHM in terms of both the quality of the modal information and network energy consumption. These OSD algorithms were feasible because most WSNs in these studies were homogeneous (sensors have the same type, storage, processing, battery power, sensing, and communication capabilities). However, when applying OSD to a HWSN that supports multi-type sensor applications, the OSD should further consider the following issues:

i. Placement performance metrics issue

In a multi-type sensor network, the orthogonality of the modal vectors cannot be exploited as in single-type sensor networks. Therefore, approaches that do not rely on the orthogonality of the modal vectors are needed.

ii. Network topology issue

Network heterogeneity allows for sophisticated operations over a larger region. As most of the energy costs come from the transmission and receiving of data packets, network topology optimization is a challenging issue for energy efficient coordination when WSNs become heterogeneous.

To the best of the authors’ knowledge, none of the existing works attempt to fulfill all the above objectives simultaneously. By employing the ratio of Modal Clarity Index (*MCI*) [23] and Mode Shape Expansion (*MSE*) [24], Jalsan et al. [25] took the information quality of the measured data collected from a HWSN into account to represent placement quality of strain and acceleration sensors. However, their work did not consider the allocation of data packets along the end-to-end path from a sensor to the base station, and therefore the optimization within HWSNs could be further improved.

In this paper, a framework is proposed to overcome the insufficiency of existing OSD techniques for HWSNs with the consideration of both placement quality and clustering issues. Typical heterogeneous devices (accelerometers and strain gauges) are considered herein since they are commonly available and have proven to be effective in detecting changes of structural properties, particularly in civil structures with low vibration amplitudes. The proposed approaches are in twofold. Firstly, a framework is proposed to optimize the layout of multi-type wireless sensors at critical locations of the structure in terms of the modal information quality and network energy consumption. Secondly, a clustering algorithm is further proposed to determine the best number of clusters in the HWSN. After the framework is laid out, a numerical verification is performed on a four-span pre-stressed reinforced concrete box-girder bridge.

The remaining sections of the paper are organized as follows: Section 2 presents the formulation of the optimization problem in HWSNs. Section 3 describes the multi-objective function and its optimization. Section 4 discusses the clustering issues in HWSN in terms of communication cost. Section 5 evaluates the performance of the proposed approach via a numerical simulation of a Heterogeneous wireless SHM system deployed on a bridge. Conclusions are drawn in Section 6.

## 2. Optimization Problem Formulation in HWSNs

The optimization for HWSN configuration is aimed to select particular physical locations for the sensors on the monitored structure such that the resulting HWSN exhibits optimal performance. To be specific, the optimization goal is set to find node locations in order to reliably identify modal information (basis for diagnosing the health of a structure) while consuming minimum energy during data collection. The formulation of optimization goal is described in the following sections.

### 2.1. Heterogeneous Sensor Placement Quality

In this study, we focus on modal identification performance based on vibration information, specifically acquired through two common types of sensors in SHM—Wireless accelerometers and strain gauges. The assessment of sensor placement quality based on modal identification accuracy has been previously studied in homogeneous WSNs. For example, the Modal Assurance Criterion (MAC) which considers the orthogonality of the modal vectors was used to evaluate the sensor placement [26]. However, in the case of heterogeneous data consisting of acceleration and strain, the orthogonality of the modal vectors is no longer available. Therefore, performance metrics such as the Modal Clarity Index (*MCI*) and Modal Relative Error (*MRE*) that do not rely on the orthogonality of the modal vectors need to be employed. A few heterogeneous sensor placement indices based on these metrics have been proposed in the literature, including the weighted sum of *MCI* and *M*RE [27], and the ratio of *MCI* and *MRE* [25]. In this work, the ratio of *MCI* and *MRE* is employed as the placement quality index to quantify the quality of the sensor placement.

#### 2.1.1. Modal Clarity Index

The objective of *MCI* is to determine the sensor locations that maximize the clarity between adjacent modes of response. The *MCI* is based on the least squares method. The best-fit amplitude matrix l is constructed using Equation (1):

(1) $$\lambda_{p,q}=\frac{\sum_{i=1}^{N_{a}+N_{s}}\alpha_{i,p}\beta_{i,q}}{\sum_{i=1}^{N_{a}+N_{s}}\alpha_{i,p}^{2}},$$

where *p* and *q* are the modes being compared, a and b are the analytical and experimental modal matrices comprising of strain measurements and acceleration measurements, respectively.

The *MCI* can be obtained as the difference between the excited mode *p* and the best fit mode *q* using Equation (2):

(2) $$MCI_{p,q}={\left[\beta_{p}-(\lambda_{p,q}\cdot \alpha_{q})\right]}^{T}[\beta_{p}-(\lambda_{p,q}\cdot \alpha_{q})],$$

The *MCI* matrix is a square matrix with dimensions equal to the modes of interest. For simplification, mean value of the sum of matrix elements are calculated as the *I**_MCI_***:

(3) $$I_{MCI}(n_{a},n_{s})=\frac{1}{m^{2}}\sum_{p=1}^{m}\sum_{q=1}^{m}I_{MCI_{p,q}},$$

where *m* is the number of measured modes, *N_a_* is the number of acceleration nodes deployed, and *N_s_* is the number of strain nodes deployed.

#### 2.1.2. Modal Relative Error

In general, it is impossible to identify all the vibration modes of a structure from measured data. Therefore, modal shape expansion (*MSE*) can be used to estimate the response of the structure at the Degrees of Freedoms (DOFs) where no sensor is equipped, from a limited number of measured DOFs [27].

The MSE method can be expressed as

(4) $$y_{\alpha }=\alpha \left[{\left(\beta^{T}\beta \right)}^{-1}\cdot \beta \right]\cdot y_{\beta },$$

in which y_a_ is the estimated response, a is the system modal matrix, b is the modal matrix for the measured DOFs, and y_b_ is the measured response of the structure.

The *MRE* can then be calculated using the estimated response and measured response via Equation (5):

(5) $$I_{MRE}=\frac{\left|y_{\alpha }-y_{\beta }\right|}{\left|y_{\beta }\right|},$$

#### 2.1.3. Placement Quality Index

The larger the corresponding *MRE*, the clearer the calculated modal information would be (and vice versa) [25]. To consider both effects of *MRE* and *MCI*, the ratio of the two metrics is defined as the placement quality index to measure the modal identification accuracy [9]:

(6) $$F_{M}=\frac{I_{MRE}}{I_{MCI}},$$

The ratio of *I_MRE_* and *I_MCI_* represents a *I_MRE_* gain per unit *I_MCI_* value. Minimizing Equation (6) yields the best combination of both measurement types. This formulation overcomes the orthogonality issue arising in heterogeneous sensors deployment and thus is used in this study.

### 2.2. Network Model

Generally, energy consumption of a typical wireless sensor node in operation mainly occurs in the phases of data acquisition, data processing, data reception, and data transmission [28]. In this study, all of these phases are considered in the energy consumption formulation for acceleration node and strain node separately. Table 1 shows the formulations of energy consumption of each part for different sensor nodes. Table 2 lists the nomenclature of parameters and their typical values used in general WSN platforms for SHM applications [29] and the following equations in this study.

**Figure 1:**
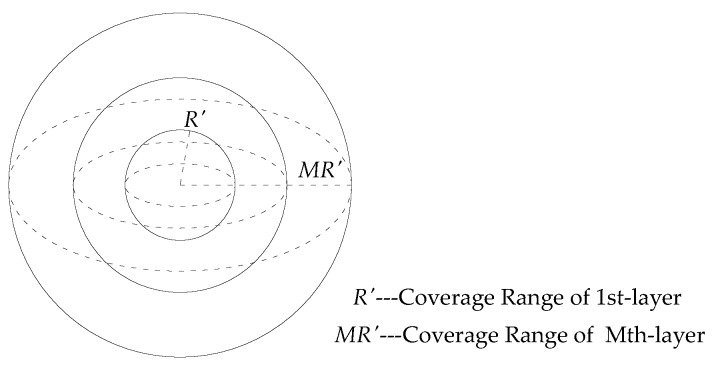
Spherical WSN model.

In order to evaluate the energy consumption of HWSNs composed of acceleration and strain nodes, a multi-layer spherical network model with a base station located in the center as shown in Figure 1 is used. Ideal communication without errors and delays is assumed. This network model, presented in the authors’ previous work [29], is now extended with heterogeneous sensor nodes deployed. If the nodes are evenly distributed in the network (in other word, obey a uniform distribution), the node density in the spherical space for acceleration *p_a_* and strain *p_s_* can be defined as follows:

(7) $$p_{a}=\frac{N_{a}}{V_{a}},p_{s}=\frac{N_{s}}{V_{s}},$$

where *V_a_* and *V_s_* are the volume of space covered by the acceleration nodes and strain nodes respectively. *N_a_* and *N_s_* have the same definition to the corresponding parameters used in Equation (1).

When considering the pre-defined sensing and routing function of sensor nodes, a multi-layer HWSN contains two main portions of energy consumption. The first portion is the energy consumed by a layer of sensor nodes due to data acquisition, processing, and information transition to the upper level. The other portion is the energy consumed by the same layer of sensor nodes for receiving data from the lower layer and passing them to the upper layer.

In the first portion, the energy consumed is estimated as:

(8) $$\begin{array}{cc} F_{E}(n,n) & =\sum_{i=1}^{N_{na}}F_{node}{\left(V_{i}\right)}_{a}+\sum_{i=1}^{N_{ns}}F_{node}{\left(V_{i}\right)}_{s}\\ & =\sum_{i=1}^{N_{na}}\{\alpha_{a}K+\beta_{a}{\left(d_{a}\right)}^{m}K+\left[\gamma_{s}+\delta_{s}{\left(d_{s}\right)}^{m}\right]K\}\\ & +\sum_{i=1}^{N_{ns}}\left\{\alpha_{s}K+\beta_{s}{\left(d_{i}\right)}^{m}K+[\gamma_{s}+\delta_{s}{\left(d_{i}\right)}^{m}]K\right\} \end{array},$$

where *N_na_* is the number of acceleration nodes in the *n*th layer; and *N_ns_* is the number of strain nodes in the *n*th layer.

Assuming the sensor nodes are uniformly deployed in the network, the number of sensor nodes in each layer can be calculated as:

(9) $$N_{na}=\frac{(3n^{2}-3n+1)R_{a}^{'}{}^{3}}{R_{a}^{3}}N_{a},\;N_{ns}=\frac{(3n^{2}-3n+1)R_{s}^{'}{}^{3}}{R_{s}^{3}}N_{s},$$

where *R_a_* and *R_s_* are the distances between layers deployed with acceleration nodes and strain nodes, respectively; *R’_a_* and *R’_s_* are the coverage ranges of all acceleration nodes and strain nodes in the network, respectively.

The expectation of data transmission distance in this process is:

(10) $$\begin{array}{cc} E(d_{i}) & =\sum_{i=1}^{N_{n}}d_{i}p_{i}=\int_{(n-1)R}^{nR}\left[x-(n-1)R\right]\frac{4\pi x^{2}}{4\pi \left\{{\left(nR\right)}^{3}-{\left[(n-1)R\right]}^{3}\right\}/3}dx\\ & =\frac{\frac{3}{2}n^{2}-n+\frac{1}{4}}{3n^{2}-3n+1}R \end{array},$$

Similarly, the energy consumed in the *n*th layer in the second portion is calculated by:

(11) $$\begin{array}{cc} F_{E}(n,n-1) & =\sum_{i=1}^{N_{na}}\left(\gamma_{a}+\delta_{a}d_{a}{}^{m}+\varepsilon_{a}\right)K+\sum_{i=1}^{N_{ns}}(\gamma_{s}+\delta_{s}d_{s}{}^{m}+\varepsilon_{s})K\\ & =\frac{(3n^{2}-3n+1)R_{a}^{'}{}^{3}}{R_{a}^{3}}N_{a}\left[\gamma_{a}+\delta_{a}{\left(\frac{\frac{3}{2}n^{2}-n+\frac{1}{4}}{3n^{2}-3n+1}R_{a}{}^{'}\right)}^{m}+\varepsilon_{a}\right]K\\ & +\frac{(3n^{2}-3n+1)R_{s}^{'}{}^{3}}{R_{s}^{3}}N_{s}\left[\gamma_{s}+\delta_{s}{\left(\frac{\frac{3}{2}n^{2}-n+\frac{1}{4}}{3n^{2}-3n+1}R_{s}{}^{'}\right)}^{m}+\varepsilon_{s}\right]K \end{array},$$

Note that the data collected in the *n*th layer data needs (*n* − 1) hops to reach the base station. By summarizing these two portion of energy consumed, the HWSN energy consumption per unit time can be expressed by:

(12) $$\begin{array}{l} F_{E}=\sum_{n=1}^{M}F_{E}(n,n)+\sum_{n=2}^{M}\left(n-1)F_{E}(n,n-1\right)\\ =\sum_{n=1}^{M}\left\{\begin{array}{l} \frac{(3n^{2}-3n+1)R_{a}^{'}{}^{3}}{R_{a}^{3}}N_{a}\left\{\alpha_{a}K+\beta_{a}{\left(\frac{\frac{3}{2}n^{2}-n+\frac{1}{4}}{3n^{2}-3n+1}R_{a}{}^{'}\right)}^{m}K+[\gamma_{a}+\delta_{a}{\left(\frac{\frac{3}{2}n^{2}-n+\frac{1}{4}}{3n^{2}-3n+1}R_{a}{}^{'}\right)}^{m}]K\right\}\\ +\frac{(3n^{2}-3n+1)R_{s}^{'}{}^{3}}{R_{s}^{3}}N_{s}\left\{\alpha_{s}K+\beta_{s}{\left(\frac{\frac{3}{2}n^{2}-n+\frac{1}{4}}{3n^{2}-3n+1}R_{s}{}^{'}\right)}^{m}K+[\gamma_{s}+\delta_{s}{\left(\frac{\frac{3}{2}n^{2}-n+\frac{1}{4}}{3n^{2}-3n+1}R_{s}{}^{'}\right)}^{m}]K\right\} \end{array}\right\} \end{array}+\sum_{n=2}^{M}\left\{\begin{array}{l} (n-1)\frac{(3n^{2}-3n+1)R_{a}^{'}{}^{3}}{R_{a}^{3}}N_{a}\left[\gamma_{a}+\delta_{a}{\left(\frac{\frac{3}{2}n^{2}-n+\frac{1}{4}}{3n^{2}-3n+1}R_{a}{}^{'}\right)}^{m}+\varepsilon_{a}\right]K\\ +(n-1)\frac{(3n^{2}-3n+1)R_{s}^{'}{}^{3}}{R_{s}^{3}}N_{s}\left[\gamma_{s}+\delta_{s}{\left(\frac{\frac{3}{2}n^{2}-n+\frac{1}{4}}{3n^{2}-3n+1}R_{s}{}^{'}\right)}^{m}+\varepsilon_{s}\right]K \end{array}\right\},$$

In this study, we assume the acceleration and strain nodes conform to the same deployment rules, that is:

(13) $$R_{a}^{'}=R_{s}^{'}=R,R_{a}=R_{s}=MR,$$

By taking Equation (13) and its typical values of the corresponding parameters into Equation (12), the energy consumption formulation for a HWSN is obtained as Equation (14):

(14) $$\begin{array}{cc} F_{E} & =\sum_{n=1}^{M}\left\{\begin{array}{l} \frac{3n^{2}-3n+1}{M^{3}}\times {\left(\frac{\frac{3}{2}n^{2}-n+\frac{1}{4}}{3n^{2}-3n+1}R\right)}^{2}\times 1.36\times 10^{-9}\times (N_{a}+N_{s})\\ +\frac{3n^{2}-3n+1}{M^{3}}\times (1.05\times 10^{-7}N_{a}+9\times 10^{-8}N_{s}) \end{array}\right\}K\\ & +\sum_{n=2}^{M}\left\{\left(n-1)\frac{3n^{2}-3n+1}{M^{3}}\left[1.8\times 10^{-7}+10^{-11}\times {\left(\frac{\frac{3}{2}n^{2}-n+\frac{1}{4}}{3n^{2}-3n+1}R\right)}^{2}\right]\times (N_{a}+N_{s}\right)\right\}K \end{array},$$

## 3. Objective Function and Solution

Trade-offs exist between modal identification accuracy and energy consumption in WSNs for SHM [21,30]. Therefore, the objective of the optimization problem herein is to determine the optimal number and location of heterogeneous sensor nodes in order to balance the amount of energy consumed and the modal identification quality in the HWSNs. The following sections describe the formulation of the multi-objective optimization function and its resolving algorithm.

### 3.1. Multi-Objective Optimization Function

A multi-objective function is formulated to describe the optimization problem as follows:

(15) $$F(F_{M},F_{E})=\phi_{M}\omega_{M}F_{M}(N_{a},N_{s},R_{a},R_{s})+\phi_{E}\omega_{E}F_{E}(N_{a},N_{s},R_{a},R_{s}),$$

where f*_M_* is the weight coefficient of modal identification, f*_E_* is the weight coefficient of energy consumption, w*_M_* is the adjustment coefficient of modal identification index, and w*_E_* is the adjustment coefficient of energy consumption. Note that each single objective function (*F_M_* and *F_E_*) has its own numerical dimension, thus adjustment coefficients are utilized to normalize the final calculation results of the multi-object function.

As discussed in Section 2.1, the ratio of MSE and *MCI* is used to formulate the objective function *F_M_* for the sensor placement quality (see Equation (6)). Meanwhile, Equation (14) in Section 2.2 is used to formulate the objective function *F_E_* for energy consumption.

### 3.2. Particle Swarm Optimization Algorithm

The optimization of the above multi-objective function for large SHM systems is computationally challenging due to the large number of DOFs. In recent years, multiple advanced optimization algorithms, such as the genetic algorithm (GA) [31], particle swarm optimization (PSO) [32], and the monkey algorithm [33], have been adopted to determine sensor optimal placements. Unlike other computation algorithms, PSO has no evolution operators, such as crossover and mutation, and therefore it has a faster convergence speed. In this study, the PSO algorithm is employed also due to its unique information diffusion capability and interaction mechanisms which enable PSO to solve complex optimization problem with high performance at low computational cost. Refer to Section 5.2 for the details of the utilization of PSO algorithm in this study.

## 4. Network Topology Optimization

HWSNs applications, such as SHM, require the equipped sensors to form a multi-hop network to collect the environmental data in real-time. Such a network typically generates a cluster-tree type topology. As most of the energy cost comes from transmitting and receiving data packets, clustering optimization become an important issue for these applications in that it benefits efficient energy coordination, thus reducing the transmission power and maximizing the network life. To this end, the topology optimization problem is considered in this section to determine an appropriate number of clusters to minimize the energy consumption after selecting the sensor number and location.

**Figure 2:**
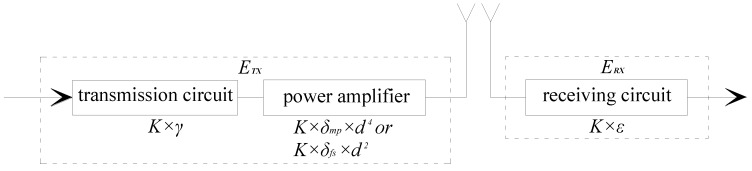
The first-order wireless communication mode.

In this study, the energy consumption model for a single sensor node is assumed to conform to the first order wireless communication mode [34] as shown in Figure 2. Note that the acceleration and strain nodes have the same radio frequency module, therefore these two types of sensors share the same communication mode.

In the data transmission process, the energy consumption of transmitting *K* bits of data to a sensor node can be expressed as:

(16) $$E_{TX}=\left\{ \begin{array}{l} K\times \gamma +K\times \delta_{mp}\times d^{4}\\ K\times \gamma +K\times \delta_{fs}\times d^{2} \end{array}\right.,$$

The energy consumption of receiving *K* bits of data from a sensor node can be expressed as

(17) $$E_{RX}=K\times \varepsilon ,$$

where *K* is the number of data packet being sent and received; γ is the energy needed to transmit a unit bit of data; ε is the energy consumption of receiving a unit bit of data; δ*_mp_* is the power amplification factor of multi-path attenuation model (*d ≥ d*_0_); δ*_fs_* is the power amplification factor of the free space model (*d < d*_0_); $d_{0}=\sqrt{d_{fs}/d_{mp}}$; *d* is the data transmission distance.

### 4.1. Two-Phase Energy Consumption Formulation

Consider a synchronized two-level cluster-tree HWSN featuring a tree-based logical topology where nodes are organized in different groups, called clusters. Each member node only interacts with its pre-defined cluster head (CH) node, and cannot be connected to other member nodes. The formulation of energy consumption in such a cluster-tree HWSN can be divided into two phases, the network initialization phase and the stable transmission phase.

Assuming that acceleration nodes, *Na*, and strain nodes, *Ns*, are randomly distributed in a *L × L × L* cube space, in which the number of CH nodes is C. Then each cluster has one CH node and (*N_a_ + N_s_*)*/C* − 1 member nodes on average. The energy consumption of CH nodes broadcasting message to member nodes is given by:

(18) $$E_{ch11}=K\times \gamma +K\times \delta_{mp}\times d^{4},$$

The determination of what cluster for a non-CH node to join is based on the strength of the signal received by the broadcast message, which is proportional to the distance *d* between the member nodes and the CH node. The energy consumption of each CH node receiving information from member nodes is given by:

(19) $$E_{ch12}=K\times \varepsilon \times \left(\frac{N_{a}+N_{s}}{C}-1\right),$$

After receiving the information, the CH node creates a Time Division Multiplexing Access (TDMA) [24] to be sent back to the member nodes, which also consumes energy. If the distance between the CH node and the member node is *d_toch_*, the energy consumption in CH is given by:

(20) $$E_{ch13}=K\times \gamma +K\times \delta_{fs}\times d_{toch}^{2},$$

According to Equations (18)–(20), the energy consumption of each CH node is given by:

(21) $$E_{ch1}=\left[\varepsilon \times \left(\frac{N_{a}+N_{s}}{C}-1\right)+2\gamma +\delta_{fs}\times d_{toch}^{2}+\delta_{mp}\times d^{4}\right]\times K,$$

Similarly, the energy consumption of a member node receiving broadcast information from the CH node is given by:

(22) $$E_{mn11}=K\times \varepsilon ,$$

The energy consumption of a member node sending a message to join the cluster is given by:

(23) $$E_{mn12}=K\times \gamma +K\times \delta_{fs}\times d_{toch}^{2},$$

The energy consumption of a member node receiving TDMA time table from the CH node is given by:

(24) $$E_{mn13}=K\times \varepsilon ,$$

Therefore, the energy consumption of each member node in an established cluster is given by:

(25) $$E_{mn1}=E_{mn11}+E_{mn12}+E_{mn13}=\left(2\varepsilon +\gamma +\delta_{fs}\times d_{toch}^{2}\right)\times K,$$

The total energy consumption for a cluster in the network initialization phase is given by:

(26) $$E_{1}=C\times \left[E_{ch1}+\left(\frac{N_{a}+N_{s}}{C}-1\right)\times E_{mn1}\right]\approx C\times E_{ch1}+\left(N_{a}+N_{s}\right)\times E_{mn1},$$

After the clusters are established, the network turns into the stable phase of data transmission. In each cluster, the member nodes send the information to the CH node, and the energy consumption of the CH node receiving this information is given by:

(27) $$E_{ch21}=K\times \varepsilon \times \left(\frac{N_{a}+N_{s}}{C}-1\right),$$

Then the CH node receives the information of the member nodes for information fusion. The energy consumption for such activity is given by:

(28) $$E_{ch22}=K\times E_{DA}\times \frac{N_{a}+N_{s}}{C},$$

where *E_DA_* is the energy consumed for fusing a unit bit of information.

Defining the distance between the CH node and base station as *d_toBs_*, the energy consumption that CH node transmits the fused information to the base station is given by:

(29) $$E_{ch23}=K\times \gamma +K\times \delta_{fs}\times d_{toBs}^{2},$$

As a result, the energy consumption of a CH node in the stable phase is given by:

(30) $$\begin{array}{cc} E_{ch2} & =E_{ch21}+E_{ch22}+E_{ch23}\\ & =\left[\varepsilon \times \left(\frac{N_{a}+N_{s}}{C}-1\right)+\gamma +\delta_{fs}\times d_{toBs}^{2}+E_{DA}\times \frac{N_{a}+N_{s}}{C}\right]\times K \end{array},$$

Each member node only sends information to the CH node. Its energy consumption is:

(31) $$E_{mn2}=K\times \gamma +K\times \delta_{fs}\times d_{toch}^{2},$$

Summarizing Equations (30) and (31), the energy consumption in the stable transmission phase is given by:

(32) $$E_{2}=C\times \left[E_{ch2}+\left(\frac{N_{a}+N_{s}}{C}-1\right)\times E_{noch2}\right]\approx C\times E_{ch2}+\left(N_{a}+N_{s}\right)\times E_{mn2},$$

Consequently, the total energy consumption for a HWSN with two-level cluster-tree is shown in Equation (33):

(33) $$E_{Total}=K\times \left\{\begin{array}{l} \varepsilon \times \left[4\left(N_{a}+N_{s}\right)-2C\right]+\left[2\left(N_{a}+N_{s}\right)+C\right]\times \delta_{fs}\times d_{toch}^{2}\\ +\gamma \times \left[3C+2\left(N_{a}+N_{s}\right)\right]+C\times \delta_{mp}\times d^{4}+C\times \delta_{fs}\times d_{toBs}^{2}\\ +E_{DA}\times \left(N_{a}+N_{s}\right) \end{array}\right\},$$

### 4.2. Optimal Clustering

In Equation (33), *d_toch_* is an uncertain value since the number of CH nodes is unknown. Therefore, we use the mathematical expectation to estimate the value of *d_toch_*.

Assuming that clusters composed of a CH node and multiple member nodes in a sphere volume are evenly distributed in the *L × L × L* cube space. In this case, for each cluster, the average volume is *L*^3^/*C*, the coverage radius is $\sqrt[{3}]{3L^{3}/4\mathrm{pC}}$, and the probability density of member nodes is *C*/*L*^3^. The expectation of *d_toch_* is:

(34) $$\begin{array}{cc} E[d_{toch}^{2}] & =\int \int \int (x^{2}+y^{2}+z^{2})\rho (x,y,z)dxdydz\\ & =\int \int \int r^{2}\rho (r,\varphi ,\theta )r^{2}\sin\varphi drd\varphi d\theta \\ & =\frac{C}{L^{3}}\int \int \int r^{4}\sin\varphi drd\varphi d\theta =0.25{\left(\frac{L^{3}}{\pi C}\right)}^{2/3}\\ & \approx \frac{0.116L^{2}}{C} \end{array},$$

With partial derivative on the number of CH nodes on both sides of Equation (34), it gives

(35) $$\frac{\partial E_{Total}}{\partial C}=3\gamma -2\varepsilon -\frac{0.233L^{2}\left(N_{a}+N_{s}\right)\delta_{fs}}{C^{2}}+\delta_{fs}d_{toBs}^{2}+\delta_{mp}d^{2},$$

After setting $\frac{\partial E_{Total}}{\partial C}=0$, it would now be possible to obtain the mathematical expression of the optimal number of CH nodes as

(36) $$C=\sqrt{\frac{0.233L^{2}\left(N_{a}+N_{s}\right)\delta_{fs}}{3\gamma -2\varepsilon +\delta_{fs}d_{toBs}^{2}+\delta_{mp}d^{4}}},$$

As can be seen in Equation (36), the optimal number of CH nodes in a HWSN is determined by the total number of sensor nodes, the distance *d* that CH nodes broadcast information to their connected member nodes, and the distance *d_toBs_* between the CH nodes and the base station.

## 5. Performance Evaluation

A numerical verification is performed on a four-span continuous reinforced concrete box-girder bridge. The optimization goal is to obtain a HWSN configuration that simultaneously minimize the network energy consumption and maximize the information quality objectives. In addition, this example also intends to show the efficiency of the proposed framework by comparing the performance of the cluster-tree topology to flat topology, which assumes that data packets are transmitted from member nodes to base station directly.

### 5.1. Simulation Setup

**Figure 3:**
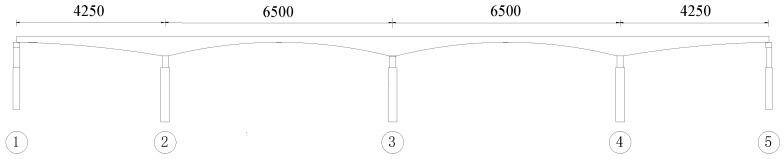
Bridge Elevation View (unit: cm).

The bridge is located in Shenzhen, China, across the Pinghu railway. It is a historical bridge currently being retrofitted. A SHM system composed of wireless acceleration and strain nodes will be implemented on the bridge to monitor its long-term vibration behaviors. The bridge has span length of 42.5 m + 2 × 65 m + 42.5 m as seen from the bridge elevation view in Figure 3.

**Figure 4:**
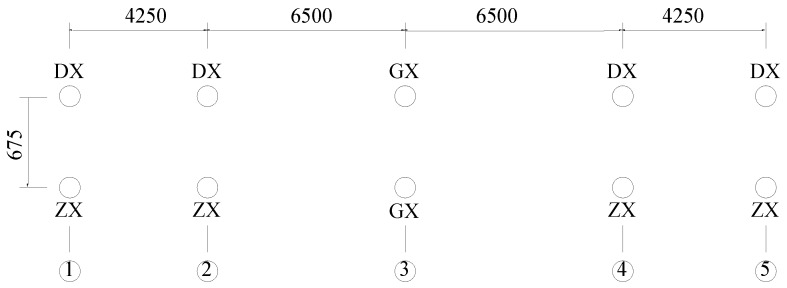
Bearing forms constraint of main bridge (unit: cm).

Bearing conditions of the bridge on a plan view are shown in Figure 4, where circles represent the double-column piers, DX represents an expansion bearing providing the bridge vertical constraint only, ZX represents an expansion bearing providing the bridge lateral and vertical constraints, and GX represents a fixed bearing.

**Figure 5:**
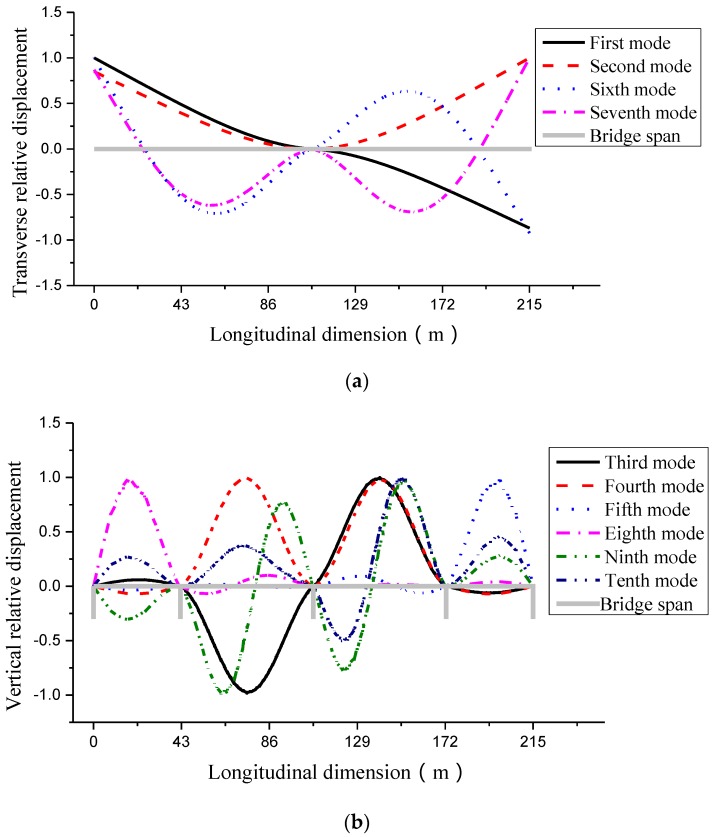
The first ten bending mode shapes of the bridge. (**a**) Lateral mode of the bridge; (**b**) Vertical mode of the bridge.

To obtain modal parameters of the bridge, a detailed finite element model was built in ANSYS. The main box girder is modeled with 9854 solid elements and 3354 nodes. In this study, the first 10 mode shapes of the main girder (exhibited in lateral and vertical modes) are considered as the targeted modes and obtained through modal analysis. The first ten modes are shown in Figure 5 with their natural frequencies are listed in Table 3.

### 5.2. Sensor Layout Optimization Results

The proposed OSD techniques are applied to determine both the optimal number of heterogeneous sensors and their best sensing locations. In order to solve the multi-objective function established in Section 5.1, it is assumed that the accuracy of the modal parameter identification and network energy consumption for this SHM system has equal significance, meaning that the weight coefficients f_M_ and f_E_ in Equation (15) are both set to be 0.5. Since the two objective values are not in the same scale of magnitude, scale factors w_R_ = 1 and w_E_ = 10^6^ are used for normalization. The MathWorks MATLAB is used to apply the PSO algorithm and carry out the calculations. The PSO parameters of initial population size, maximum number of iterations, and particle velocity, are set to be 300, 150, and [−3, 3], respectively. The PSO is run for 10 times to generate the network layouts. The objective function values, accelerometers, strain gauges, and coverage ranges of the layouts obtained in the ten cases are listed in Table 4.

**Figure 6:**
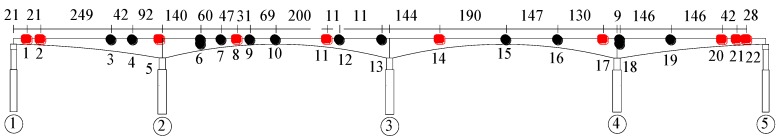
Sensor deployment on the bridge girder.

**Figure 7:**
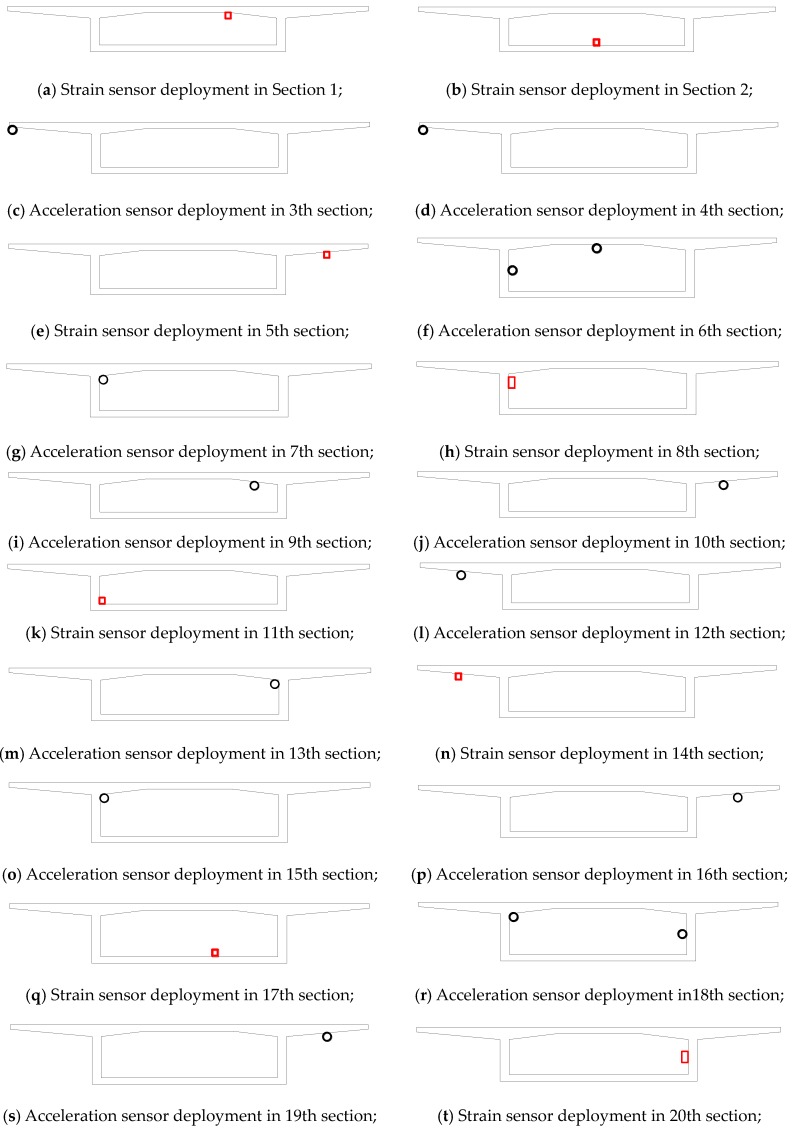

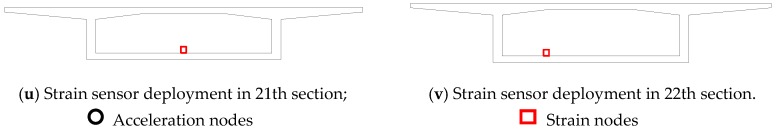
Sensor deployment on bridge cross sections.

Herein, the mean values are used as the optimal sensor layout deployed on the bridge plan. The optimal number of accelerometers, strain gauges, and coverage range are 14, 10, and 96.7 m, respectively. The sensor placement layout found from the analysis is shown in Figure 6, where black circles indicate wireless accelerometers and red rectangles represent the wireless strain gauges, respectively. Numbers 1~22 listed under the bottom of the bridge represent selected cross sections that are equipped with sensors. Numbers listed above the bridge represent longitudinal distance (cm) between sensors. The results showed non-symmetric sensor configurations in the longitudinal direction. Detailed sensor locations on each section are shown subsequently in Figure 7.

### 5.3. Clustering Optimization Results

The study herein is aimed to determine the best data transmission path of the HWSN based on the sensor configuration from initial optimization results, and to compare the performance of network topology effects on energy consumption. The related network parameters on the bridge monitoring system are listed in Table 5.

Since the probability density ρ(*x*, *y*, *z*) of the CH nodes is the reverse of the HWSN space *V*, the distance expectation *d_toBs_* is calculated using Equation (35):

(37) $$\begin{array}{cc} E[d_{toBs}^{2}] & =\int \int \int \left[{\left(x-107.5\right)}^{2}+y^{2}+{\left(z-6.875\right)}^{2}\right]\rho (x,y,z)dxdydz\\ & =\frac{1}{V}\int \int \int \left[{\left(x-107.5\right)}^{2}+y^{2}+{\left(z-6.875\right)}^{2}\right]dxdydz\\ & =7.8\times 10^{3}m^{2} \end{array},$$

**Figure 8:**
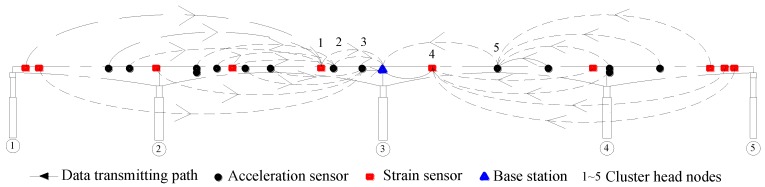
Cluster-tree topology configuration.

Subsequently, taking these parameter values into Equation (36), the optimal number of clusters is obtained as five. That is, the wireless sensors selected to monitor the bridge are divided into five clusters. Nevertheless, finding specific member nodes that result in best data transmission path in a large-scale HWSN with multiple clusters is a quite complex problem. In this study, for fast optimization, the nodes close to the base station are assumed to be selected as CH nodes and the member nodes in each cluster are allocated as even as possible. Figure 8 shows the cluster-tree based HWSN topology on the bridge in a 2-D configuration. Each cluster has four or five member nodes. The dotted line indicates the data transmission path.

In order to evaluate the effectiveness of clustering optimization, the simulation results using the proposed method are compared to those obtained by a flat network topology (one layer). According to Equation (14) derived in Section 2.2, the network energy consumption for both scenarios is calculated and shown in Table 6.

In the data transmission path produced by the cluster-tree HWSN topology, the coverage radius is increased by 28.3% compared to that of the flat topology. In addition, it is also found that the energy consumption of the sensors is significantly lower than that of the flat topology (33.3%). This reduction is expected since, although the coverage radius increases, the transmission distance of data packets is reduced in a cluster-tree routing path, which indicates that the energy consumption depends not only on the coverage radius of network, but also other factors such as the data transmission distance and the amount of transmitted data packets. Since there exists a linear relationship between energy consumption and the square of data transmission distance, the energy saving in a cluster-tree topology can be achieved by reducing total data transmission distance through suitable clustering mechanism, and, therefore, it is independent of the network size.

It should be emphasized that the optimization of network topology in this study only involves a fast selection of CH nodes according to the algorithm proposed. Further determination of specific member nodes in each cluster will lead to another complex problem which is out of the scope of this paper. In the expense of larger amount of computation time, the data packets transmission path shown in this study could be further optimized. However, the OSD framework proposed is applicable to other monitoring scenarios that the deployment of multi-type wireless sensors is based on the principle of modal information quality and minimum of energy consumption.

## 6. Conclusions

The increasing interest in employing HWSNs for SHM applications requires an efficient sensor placement methodology. In this study, first, an OSD framework for HWSNs is proposed. A multi-objective layout optimization problem is presented and resolved using the PSO algorithm to determine the trade-off between modal information quality and energy consumption with the consideration of the application requirements. Furthermore, a clustering optimization approach is proposed to conserve extra energy through selecting appropriate CH nodes in multi-hop HWSNs. A continuous-span bridge is used as an example to evaluate the performance of the proposed approach for designing a wireless SHM system comprised of acceleration nodes and strain nodes on the bridge.

The proposed optimization approaches can effectively determine the best number and location of heterogeneous wireless sensors for the purpose of SHM. Simulation results demonstrate that a set of strategically positioned heterogeneous sensors can maintain an optimal balance between the modal information accuracy and energy consumption. With the determined sensor deployment configuration, the proposed clustering optimization approach can be easily implemented to further conserve energy by selecting appropriate CH nodes in a multi-hop HWSN.

## Figures and Tables

**Table 1:** The energy consumption functions of each unit about different sensor nodes.

Energy Consumption	Wireless Accelerometer Node	Wireless Strain Gauge Node
Data acquisition	*C_ca_* = a*_a_K*	*C_cs_* = a*_s_K*
Data processing	*C_pa_* = b*_a_d_a_^m^K*	*C_ps_* = b*_s_d_s_^m^K*
Data communication	*C_ta_* = (ga + d*_a_*d*_a_^m^*)*K*	*C_ts_* = (g*_s_* + d*_s_*d*_s_^m^*)*K*
Data receiving	*C_ra_* = e*_a_K*	*C_rs_* = e*_s_K*

**Table 2:** Nomenclature and typical values.

Parameter	Definition	Value
α_a_	energy consumption related to data collecting about accelerometer	60 × 10^−9^ J/bit
β_a_	energy consumption related to data processing about accelerometer	45 × 10^−9^ J/bit
γ_a_	energy consumption related to the transmission distance about accelerometer	45 × 10^−9^ J/bit
δ_a_	energy consumption related to the transmission distance about accelerometer	10 × 10^−12^ J/bit.m^2^
ε_a_	energy consumption related to data receiving about accelerometer	135 × 10^−9^ J/bit
*d*_a_	average distance between all nodes in a network layer to the base station or cluster head node in upper network layer	/
α_s_	energy consumption related to data collecting about strain sensor	45 × 10^−9^ J/bit
β_s_	energy consumption related to data processing about strain sensor	1.35 × 10^−9^ J/bit
γ_s_	energy consumption related to data transmitting about strain sensor	45 × 10^−9^ J/bit
δ_s_	energy consumption related to the transmission distance about strain sensor	10 × 10^−12^ J/bit.m^2^
ε_s_	energy consumption related to data receiving about strain sensor	135 × 10^−9^ J/bit
*d*_s_	average distance between all nodes in a network layer to the base station or cluster head node in upper network layer	/
*K*	amount of data per second transmitting	200

**Table 3:** The first ten natural frequencies of the bridge.

Mode	Frequency (Hz)	Modal Shape
1	0.669	lateral
2	0.746	lateral
3	3.291	vertical
4	3.541	vertical
5	4.051	vertical
6	4.367	lateral
7	5.118	lateral
8	5.370	vertical
9	7.935	vertical
10	8.450	vertical

**Table 4:** Results of the PSO running 10 times.

Number	1	2	3	4	5	6	7	8	9	10	Mean
Function Value	154	188	173	138	157	185	122	99	162	63	144.1
N1 (Accelerometer)	14	14	15	14	15	13	15	13	13	14	14
N2 (Strain Gauge)	10	10	9	10	9	11	9	11	11	10	10
R(m) (Coverage Range)	103	97	89	98	99	100	91	101	100	102	96.7

**Table 5:** Network parameters.

Parameter	Value
HSWN Space V	(0, 0, 0)~(215, −3.12, 13.75)
Accelerometer	14
Strain Sensor	10
Base Station Location	(107.5, 0, 6.875)
γ	45 × 10^−9^ J/bit
ε	135 × 10^−9^ J/bit
δ_fs_	10 × 10^−12^ J/bit/m^2^
δ_mp_	0.0013 × 10^−12^ J/bit/m^2^
d	100 m

**Table 6:** Comparison of clustering and non-clustering.

Network Topology	Energy Consumption (J)	Coverage Radius of Network (m)		
		First Layer	Second Layer	Entire Network
Flat	0.0280	—	—	105.4
Cluster-tree	0.0210	32.7	102.5	135.2
Difference (%)	33.3	—	—	28.3
